# Multiple Group I Introns in the Small-Subunit rDNA of *Botryosphaeria dothidea*: Implication for Intraspecific Genetic Diversity

**DOI:** 10.1371/journal.pone.0067808

**Published:** 2013-07-02

**Authors:** Chao Xu, Chunsheng Wang, Xinyao Sun, Rong Zhang, Mark L. Gleason, Tanaka Eiji, Guangyu Sun

**Affiliations:** 1 Key Laboratory of Crop Stress Biology in Arid Areas, College of Plant Protection, Northwest A&F University, Yangling, Shaanxi, China; 2 Department of Plant Pathology and Microbiology, Iowa State University, Ames, Iowa, United States of America; 3 Ishikawa Prefectural University, Ishikawa, Japan; University of Florida, United States of America

## Abstract

*Botryosphaeria dothidea* is a widespread and economically important pathogen on various fruit trees, and it often causes die-back and canker on limbs and fruit rot. In characterizing intraspecies genetic variation within this fungus, group I introns, rich in rDNA of fungi, may provide a productive region for exploration. In this research, we analysed complete small subunit (SSU) ribosomal DNA (rDNA) sequences of 37 *B. dothidea* strains, and found four insertions, designated Bdo.S943, Bdo.S1199-A, Bdo.S1199-B and Bdo.S1506, at three positions. Sequence analysis and structure prediction revealed that both Bdo.S943 and Bdo.S1506 belonged to subgroup IC1 of group I introns, whereas Bdo.S1199-A and Bdo.S1199-B corresponded to group IE introns. Moreover, Bdo.S1199-A was found to host an open reading frame (ORF) for encoding the homing endonuclease (HE), whereas Bdo.S1199-B, an evolutionary descendant of Bdo.S1199-A, included a degenerate HE. The above four introns were novel, and were the first group I introns observed and characterized in this species. Differential distribution of these introns revealed that all strains could be separated into four genotypes. Genotype III (no intron) and genotype IV (Bdo.S1199-B) were each found in only one strain, whereas genotype I (Bdo.S1199-A) and genotype II (Bdo.S943 and Bdo.S1506) occurred in 95% of the strains. There is a correlation between *B. dothidea* genotypes and hosts or geographic locations. Thus, these newly discovered group I introns can help to advance understanding of genetic differentiation within *B. dothidea*.

## Introduction

Group I introns, a class of RNAs with autocatalytic activity that was initially discovered in *Tetrahymena*, are also known as ribozymes [1]. Their characteristic RNA folds consist generally of 10 conserved paired elements (P1 to P10) that can help catalyze a two-step, self-splicing transesterification reaction resulting in intron release and ligation of the exons [2]. On the basis of their primary and secondary structural features, group I introns are currently divided into five subgroups (IA to IE), some of which are further subdivided (IA1, IC3, etc.) according to the presence/absence of peripheral paired elements [3], [4]. Additionally, a minority of the reported group I introns have open reading frames (ORFs) for encoding homing endonucleases (HEs), which fall into these four families - LAGLIDADG, GIY-YIG, His-Cys box and NHN - based on the conserved motifs [5]. Frequently found in the rDNA region of fungi, these highly variable group I introns have been used increasingly as a molecular genotyping tool to explore the population diversity of this lineage [6], [7].

The filamentous ascomycete *Botryosphaeria dothidea* (Moug. : Fr.) Ces. & De Not. was designated as the type species by Barr [8] and Slippers *et al*. [9]. Ubiquitous and cosmopolitan, it has been found in most tropical and warm temperate regions as a pathogen on dozens of economically important shrub and tree fruit crops including apple, pistachio, peach, grape and blueberry, causing symptoms such as die-back, stem and shoot blight, gummosis, canker and fruit rot [10], [11], [12], [13], [14]. Along with its broad range of host species, symptoms and geographic occurrence, *B. dothidea* displays high levels of diversity. Moreover, Ma et al. [15] identified several populations of *B. dothidea* collected from pistachio and other plant hosts in California based on their differences in growth rate, virulence on hosts and fungicide sensitivity. Peng et al. [16] separated the isolates causing apple ring rot into two distinct groups by ITS sequence and ISSR diversity analysis.

Previous studies have integrated several molecular methods (e.g., RAPD, MP-PCR and Phylogenetics) to determine genetic variation and the population structure of *B. dothidea* [17], [18]. In the present study, complete small-subunit (SSU) rRNA genes of 37 strains of this fungus were amplified to seek insertions as the first step toward exploiting a new polymorphic marker for *B. dothidea*. To characterize the nature of these SSU insertions, we performed sequence alignments and BLAST analysis, and eventually inferred their secondary structures. Finally, we investigated variable distribution of the insertions within our *B. dothidea* isolates to assess their roles as indicators for intraspecific genetic diversity.

## Materials and Methods

### Fungal Strains and Extraction of Genomic DNA

Of 37 strains of *Botryosphaeria dothidea* which were studied, 32 originated from various plant hosts throughout China. The remaining five strains, regarded as the out-group, were provided by International Collection of Microorganisms from Plants (ICMP) in New Zealand, Centraalbureau voor Schimmelcultures (CBS) in the Netherlands, and Fruit Tree Research Experiment Station of the Ministry of Agriculture, Forestry and Fisheries (MAFF) in Japan. Detailed information about strains is provided in Table 1. All strains were preserved in glycerol at −80°C and sub-cultured on potato dextrose agar (PDA) before extraction of genomic DNA.

**Table 1:** *Botryosphaeria dothidea* strains examined in this study and distribution of introns within them.

			Presence (+) or Absence (−) of insertions in SSU (S)				
Strain	Host	Locality	Bdo.S943	Bdo.S1199-A	Bdo.S1199-B	Bdo.S1506	Genotype
PG-HN-3	Apple	China	−	+	−	−	I
PG-SX-3	Apple	China	−	+	−	−	I
PG-SX-4	Apple	China	−	+	−	−	I
MAFF 645001	Apple	Japan	−	+	−	−	I
YS-SX-1	Poplar	China	−	+	−	−	I
YS-SX-4	Poplar	China	−	+	−	−	I
YS-SX-5	Poplar	China	−	+	−	−	I
YS-SX-7	Poplar	China	−	+	−	−	I
ICMP 8019	*Populus nigra*	New Zealand	−	+	−	−	I
SL-SX-2	Pomegranate	China	−	+	−	−	I
SL-SX-3	Pomegranate	China	−	+	−	−	I
SL-SX-4	Pomegranate	China	−	+	−	−	I
TS-SX-2	Peach	China	−	+	−	−	I
TS-SX-4	Peach	China	−	+	−	−	I
TS-SX-5	Peach	China	−	+	−	−	I
ZS-SX-1	Jujube	China	−	+	−	−	I
ZS-SX-3	Jujube	China	−	+	−	−	I
ZS-SX-4	Jujube	China	−	+	−	−	I
HT-SX-1	Walnut	China	−	+	−	−	I
HT-SX-2	Walnut	China	−	+	−	−	I
LIU-SX-5	Willow	China	−	+	−	−	I
PT-SX-2	Grapevine	China	−	+	−	−	I
SZ-SX-1	Persimmon	China	−	+	−	−	I
MAFF 410826	*Prunus* sp.	Japan	−	+	−	−	I
PG-HB-1	Apple	China	+	−	−	+	II
PG-SHX-1	Apple	China	+	−	−	+	II
PG-JS-2	Apple	China	+	−	−	+	II
PG-SD-1	Apple	China	+	−	−	+	II
PG-SD-2	Apple	China	+	−	−	+	II
PG-HN-1	Apple	China	+	−	−	+	II
MAFF 645002	Apple	Japan	+	−	−	+	II
LS-SX-1	Pear	China	+	−	−	+	II
LS-SX-3	Pear	China	+	−	−	+	II
LS-SX-4	Pear	China	+	−	−	+	II
LS-SX-6	Pear	China	+	−	−	+	II
PG-HN-4	Apple	China	−	−	−	−	III
CBS 115476	*Prunus* sp.	Switzerland	−	−	+	−	IV

For DNA extraction, mycelium of the tested strains was first transferred from culture plates to 1.5-ml EP tubes and then ground with a tissue homogenizer. Subsequently, an aliquot of macerated mycelium was suspended in 600 µl of 2% CTAB buffer and incubated for at least 30 min at 65°C. Finally DNA was extracted using a phenol-chloroform extraction process and resuspended in 50 µl of 1×TE buffer (10 mM Tris-HCl, pH = 8.0; 1 mM EDTA).

### PCR Amplification and Sequencing

In order to obtain the entire SSU rDNA sequence, the primer pair SSU-F (5′-GCTTGTCTCAAAGATTAAGCC-3′) and SSU-R (5′-CAAAGCAACAGAGGTAGGTAC-3′) was designed according to the conserved 18S regions of species available in GenBank that were closely related to *B. dothidea*. PCR was performed in a reaction mixture of 25 µl containing approximately 10–30 ng fungal genomic DNA, 10×Taq buffer with (NH_4_)_2_SO_4_, 1.5 mM MgCl_2_, 0.2 µM of each dNTP, 5 pmol of each primer, 1 U Taq polymerase and sterile ultrapure water. The PCR conditions consisted of an initial denaturation at 94°C for 2 min, followed by 35 amplification cycles of denaturation at 94°C for 35 s, annealing at 49°C for 30 s, elongating at 72°C for 3 min and then one final step at 72°C for 10 min.

ITS1 (5′-TCCGTAGGTGAACCTGCGG-3′) and ITS4 (5′-TCCTCCGCTTATTGATATGC-3′) were used to amplify the entire internal transcribed spacer (ITS) region [19]. The PCR reaction system was the same as above and the PCR conditions consisted of an initial denaturation at 94°C for 2 min, followed by 35 amplification cycles of denaturation at 94°C for 35 s, annealing at 55°C for 30 s, elongating at 72°C for 1 min and one final step at 72°C for 10 min.

PCR products (5 µl) were electrophoresed in 1% agarose gel in the presence of EZVISION ONE, DNA DYE & BUFFER, and visualized under UV light. Acquired unpurified PCR products with clear target bands were sent directly to Sangon Biotech (Shanghai) Co, Ltd. for sequencing.

### Sequence Analysis

To locate intron insertion positions, the obtained sequence data of amplicons were aligned with a reference SSU sequence without insertions by using Clustal X 2.0 software [20]. The same software was also used to investigate polymorphisms among introns at the same site. Double sequence alignment among the introns of different sites was performed with the online bl2seq programme of Specialized BLAST (algorithm: somewhat similar sequences), and similarity searches of the introns were done on the BLASTN programme (http://blast.ncbi.nlm.nih.gov/Blast.cgi) to preliminarily determine their attributes.

### Characterization of the Introns

Mfold Web Server (http://mfold.rna.albany.edu/?q=mfold) [21] was used to predict and estimate the RNA folding structures of the insertions. These preliminary secondary structural models were then manually modified with the software RnaViz 2.0 according to pre-existing structural conventions for group I introns [22], [23], [24]. Finally, the models were manually optimized based on previous studies of group 1 introns [25], [26].

Open reading frame (ORF) prediction and translation were performed by the online programme GeneMark.hmm (http://exon.biology.gatech.edu/eukhmm.cgi) [27]. The BLASTP program was used for protein similarity searches (http://blast.ncbi.nlm.nih.gov/Blast.cgi). Alignment of amino acid sequences was carried out by Clustal X 2.0 and edited by BioEdit version 5.0.9 [28].

## Results

### Nucleotide Structures of *B. dothidea* SSU rDNA

**Figure 1:**
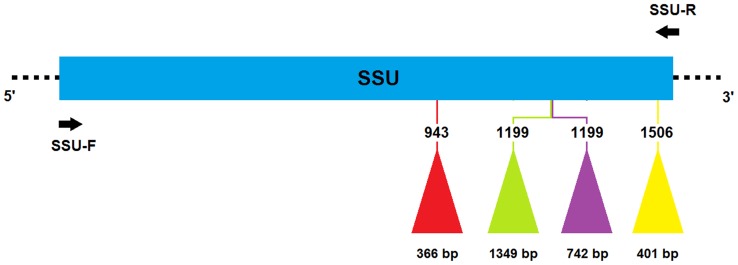
Primary structure of the SSU rDNA in *Botryosphaeria dothidea*. The entire SSU rDNA is shown by the blue rectangle. The triangles in red, green, purple and yellow respectively indicate four different introns. The position and length of each intron are given separately above and below the triangles. The insertion sites correspond to the 16S rRNA of *E. coli* J01859. Primers are presented by black arrows.

The SSU rDNA of 37 *B. dothidea* strains were amplified to investigate the presence or absence of insertions. The expected size of these amplicons without insertion was 1835 bp. As detected by agarose gel electrophoresis, however, actual sizes were 1800, 2600 or 3200 bp. In order to precisely determine the sites and sizes of insertions, all amplicons with a larger size than the expected 1835 bp were sequenced. Fragments 3200 bp in size contained 1349-bp insertions at position 1199 (intron insertion sites correspond to the 16S rRNA of *E. coli* J01859 throughout this paper). The 2600-bp fragments revealed 366- and 401-bp insertions at positions 943 and 1506, respectively, except for one with a 742-bp insertion at position 1199 (Figure 1).

In total, sequences of 11 insertions of 366 bp, 24 insertions of 1349 bp, one insertion of 742 bp and 11 insertions of 401 bp were obtained. Respective alignments of the four categories of insertions above showed 100% identity, and they were named Bdo.S943, Bdo.S1199-A, Bdo.S1199-B and Bdo.S1506, respectively, after host and site of occurrence on the genome site. Sequences of the four insertions were deposited in GenBank under the accession numbers KC702791 (Bdo.S943), KC702792 (Bdo.S1199-A), KC702793 (Bdo.S1199-B), KC702794 (Bdo.S1506).

### Sequence Analysis of Insertions

**Figure 2:**
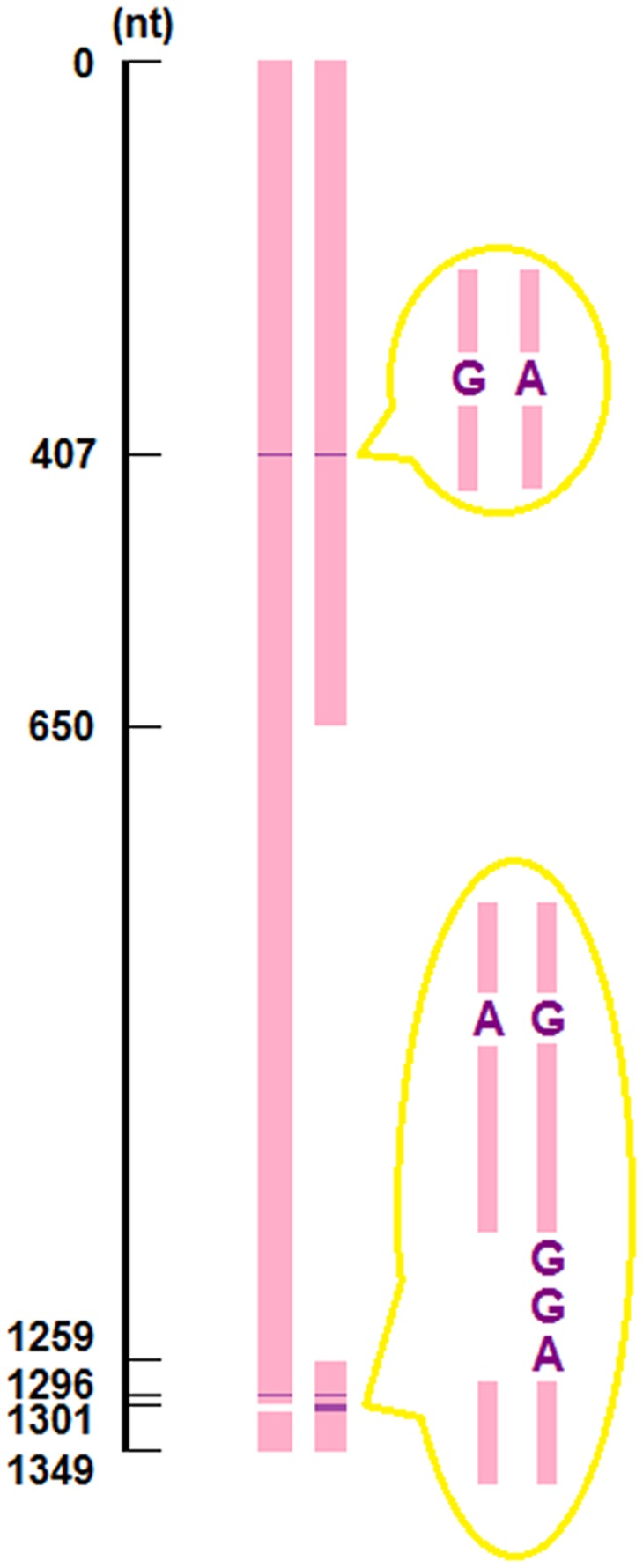
Pairwise alignment between Bdo.S1199-A and Bdo.S1199-B. The two bars refer to the RNA sequences of Bdo.S1199-A (left) and Bdo.S1199-B (right), respectively. The pink bars correspond to the identical regions of two sequences. The purple stripes on the bars point to SNPs or insertion position whereas the white areas represent deletions.

Pairwise comparison was made among the four insertions, which divided them into two sets. A homology (70% identity) was revealed between Bdo.S943 and Bdo.S1506, indicating that they belonged to the same class. In contrast, Bdo.S1199-A and Bdo.S1199-B were highly similar in sequence (99% identity), although unlike Bdo.S1199-A, Bdo.S1199-B lacked a 610-nt-long region ranging from 650∼1259 nt and had a triplet of nucleotides GGA inserted at position 1301; in addition, an A/G polymorphism at position 407 and a G/A polymorphism at position 1296 were found (all positions refer to Bdo.S1199-A) (Figure 2).

BLASTN analyses of these insertion sequences were performed in order to identify their nature subsequently. The best match (79% query coverage and 91% identity) obtained for Bdo.S943 corresponded to an entry (GenBank accession number: AY307355) described as the group IC1 intron in *Scytalidium dimidiatum* (teleomorph: *Neoscytalidium dimidiatum*) [25]. Although non-annotated subject sequences belonging to *N. dimidiatum* (>90% identity) of Bdo.S1506 made it difficult to directly define this insertion, the similarity of Bdo.S1506 to Bdo.S943 led us to regard it as a group IC1 intron as well. In addition, we used the entire 1349-bp Bdo.S1199-A for searching in GenBank and obtained four blast hits of high similarity (19–22% query coverage and 82–83% identity), all of which were described as ∼390-bp group IE introns of *S. dimidiatum* (GenBank accession numbers: AY307357, AF258605, AF258604, AF258603) [26]. However the low query coverage suggested that Bdo.S1199-A was composed of two parts: the first domain (from 1 to about 400 nt) corresponding to group IE intron and the second domain (the remaining ∼1000 bps) containing a homing endonuclease gene (HEG) [5]. The BLASTN result of Bdo.S1199-B was the same as Bdo.S1199-A, which agreed with their high sequence similarity.

### Secondary Structures of Bdo.S943 and Bdo.S1506

**Figure 3:**
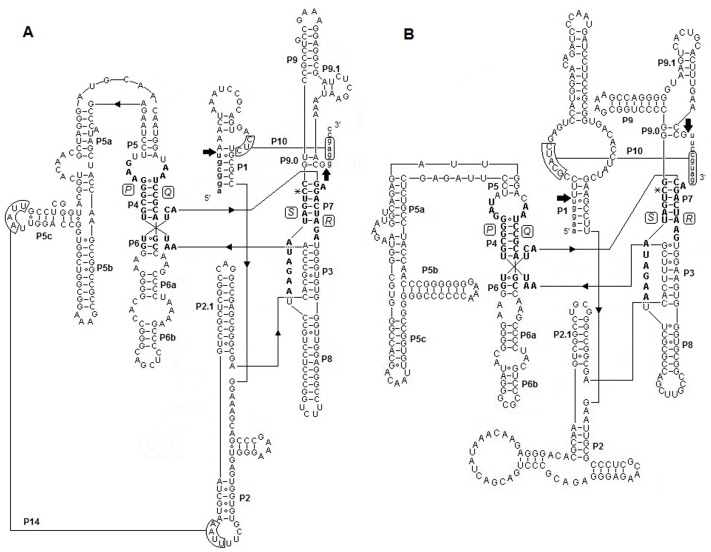
Putative secondary structures of group IC1 introns of *B. dothidea*. A. Secondary structure of Bdo.S943. B. Secondary structure of Bdo.S1506. Putative Watson-Crick and wobble base pairs are shown by lines and hollow circles, respectively. Capital and small letters represent intron and exon nucleotides, respectively. The boldface letters indicate four conserved core sequence elements P, Q, R and S. Arrows point to the 5′ and 3′ splice sites. The guanosine cofactor binding-sites are marked with *.

Compared and aligned with group IC1 introns described previously [25], [29], the predicted secondary structure of Bdo.S943 was constructed (Figure 3A). It was characterized by the requisite U (the last nucleotide of the upstream exon) and G (the last nucleotide of the intron) residues at the 5′ and 3′ splice sites. The four conserved elements P (106–117), Q (220–229), R (267–280) and S (318–329) were used to build the core pairing regions P4 and P7. The P10 stem was composed of a partial P1 region complementary to the first nucleotides of the 3′ exon. Two long-distance pairing P3 (forming a pseudoknot) and P14 may have contributed to stabilizing the tertiary structure of the intron. A G274-C328 pair within P7 corresponded to the guanosine cofactor binding site involved in the self-splicing of group I introns [30]. The catalytic core was defined by a set of structurally conserved elements, including P3–P8. In addition, Bdo.S943 contains an extended P5 domain (P5a–P5c) and the bulged nucleotide in P7 is an A [22]. GC of the 3′ end of the Q element and GU the 5′ end of the R element are paired in P6 [31]. The nucleotides A106, A107 and U108 are located in segments J3/4 of Bdo.S943 [4], [32]. All observations confirmed classification of Bdo.S943 as a group IC1 intron.

Analogously, we constructed the secondary structure of Bdo.S1506 by consulting Bdo.S943 (Figure 3B). The four conserved elements P (152–163), Q (258–267), R (300–313) and S (345–356) were identified along the sequence of Bdo.S1506. A G307-C355 pair within P7 corresponded to the G-binding position. The GU-CG pair of P6 and the A152, A153, U154 in J3/4 were in accord with Bdo.S943. Similarly, an extended P5 domain (P5a–P5c) and the bulged nucleotide A in P7 were present in Bdo.S1506. As a result, Bdo.S1506 was also identified as a group IC1 intron.

### Characterization of Bdo.S1199-A and Bdo.S1199-B

**Figure 4:**
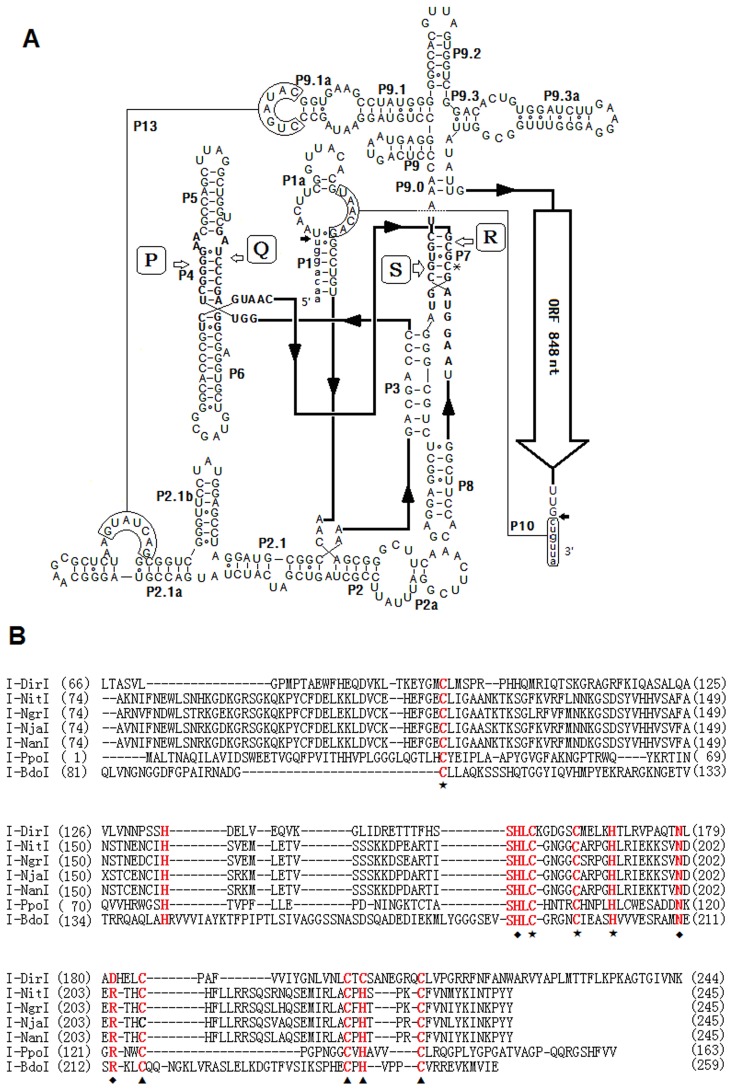
Description of Bdo.S1199-A. A. Secondary structure of Bdo.S1199-A. Capital and small letters represent intron and exon nucleotides, respectively. Putative Watson-Crick and wobble base pairs are shown by lines and hollow circles, respectively. The boldface letters indicate four conserved core sequence elements P, Q, R and S. Arrows point to the 5′ and 3′ splice sites. The guanosine cofactor binding-sites are marked with *. The open reading frame (ORF) for a His-Cys box homing endonuclease is located at the 3′ terminal of the intron. B. Alignment of partial sequences of His-Cys box homing endonucleases. The putative homing endonuclease sequence in *B. dothidea* (I-BdoI) is aligned with six His-Cys box homing endonucleases of demonstrated activity. Conserved residues are indicated in red. ★ designates residues involved in the first zinc coordination. ▴ designates residues involved in the second zinc coordination. ♦ designates active site residues.

We predicted the secondary structure of Bdo.S1199-A by its first domain (Figure 4A) in reference to the previously described group IE introns [33]. The requisite U and G residues at the 5′ and 3′ splice sites were present and the four conserved elements P (149–159), Q (178–187), R (211–224) and S (252–263) were identified. The general base pair segments of P1∼P10 were constructed; in addition, an extra segment, P13, was believed to contribute to stabilizing the auto-catalytic core [26]. Compared with IC1 introns, Bdo.S1199-A had a non-expanding P5 and expanding P2 and P9 regions. A G259-C221 pair of P7 was identified as the binding site of the guanosine-cofactor. The element P (J3/J4) started with GG and the 9th nucleotide of element R and its counterpart, the 10th nucleotide of the S element, were C and G respectively. The 2A in the J4/5 segments and the A at position J6/7 were drawn. The evidence therefore indicated that Bdo.S1199-A belongs to subgroup IE [4]. In addition, the putative HE-coding region did not invade common non-critical regions (i.e. terminal loops, such as L1, L2, L6 and L8) [34], but inserted into the 3′ terminal nucleotides of Bdo.S1199-A outside the true secondary structure.

On the second domain of Bdo.S1199-A, we identified a protein sequence of 259 amino acids which began with an AUG initiator codon at position 478 and ended with a UAA terminal codon at position 1323. BLASTP analysis indicated similarity to the His-Cys box HE of *S. dimidiatum* (GenBank ID: AAP73783), with an E-value of 0.014. Amino acid sequence alignment (Figure 4B) of the putative protein with six well-known His-Cys box HEs (Table 2) revealed the presence of eight typical conserved residues involved in two zinc coordination [35]. The first bound zinc ion was coordinated by a cluster of three Cys ligands and one His ligand, Cys_100_ (90X) Cys_191_ (4X) Cys_196_ (4X) His_201_. Furthermore, the motif coordinating the second zinc ion corresponded to the sequence Cys_216_ (26X) Cys_243_ X His_245_ (3X) Cys_249_. The two conserved zinc-binding motifs characterized by a series of Cys and His residues were named His-Cys box. Moreover, additional conserved residues were regarded as the catalytic sites of the enzyme. Bdo.S1199-A, therefore, proved to be a group IE intron containing a putative His-Cys box HEG, regardless of whether the gene can still be expressed.

**Table 2:** His-Cys box homing endonucleases used for alignment.

Abbr. ofenzyme	Organism	No. of AA	Type	GenBank ID
I-PpoI	*Physarum polycephalum*	163	ACT	1EVW_ A
I-NgrI	*Naegleria gruberi*	245	ACT	CAA55084
I-NjaI	*N. jamiesoni*	245	ACT	AAB71747
I-NanI	*N. andersoni*	245	ACT	CAA55086
I-NitI	*N. italica*	245	ACT	AAB71746
I-DirI	*Didymium iridis*	244	ACT	CAI83766
I-BdoI	*Botryosphaeria dothidea*	259	FL	AGJ76538

Note: ACT: Enzyme with demonstrated activity; FL: predicted full-length ORF.

According to the previous result of pairwise alignment, we determined that Bdo.S1199-B presented the same secondary structure as Bdo.S1199-A in spite of the large sequence difference in their so-called protein-coding regions. Deletion of a large fragment in the Bdo.S1199-B made it unlikely that an ORF would occur on its terminal ∼350-nt area. Therefore, see Figure 4A to view the proposed secondary structure of Bdo.S1199-B.

### Genotyping of *B. dothidea* Strains

**Figure 5:**
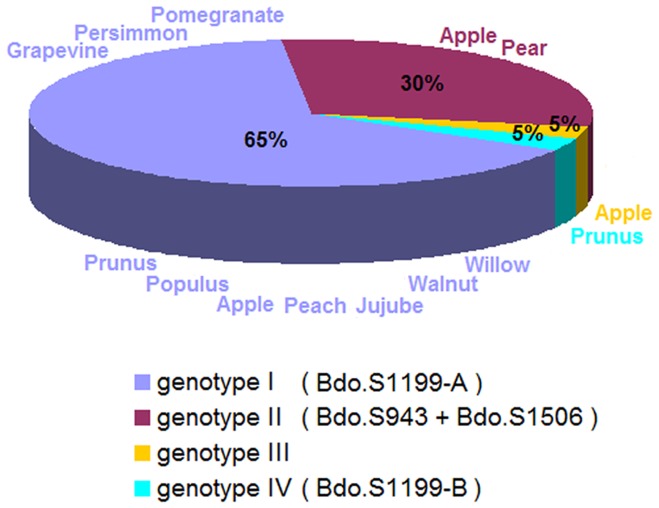
Distribution of the different introns across hosts and populations. Four different colours represent four populations divided by presence or absence and types of introns. Hosts for each population are listed in the corresponding colours. Ratios of the four populations are marked at the relevant position of the pie chart.

Thirty-six strains of *B. dothidea* each possessed one or more of the introns Bdo.S943, Bdo.S1199-A, Bdo.S1199-B and Bdo.S1506 (Table 1). Four genotypes based on the frequency and distribution of introns were described to discriminate within the surveyed population of 37 *B. dothidea* strains: 24 strains (genotype I, 65%) possessed only Bdo.S1199-A, Bdo.S943 and Bdo.S1506 coexisted in the 18S region of 11 *B. dothidea* strains (genotype II, 30%), and of the remaining two strains (5%), one (genotype III) had no introns and the other (genotype IV) contained Bdo.S1199-B (Figure 5). Moreover, all of genotype I strains were recovered from apple, *Populus* spp., pomegranate, peach and various other hosts in China, Japan and New Zealand. The strains of genotype II originated only from apple and pear in China and Japan. Genotypes III and IV, each of which contained only one strain, were respectively from apple in China and *Prunus* sp. in Switzerland.

Two single nucleotide polymorphisms (SNPs) were found by scanning ITS sequences of all the *B. dothidea* strains studied: a T/C polymorphism at position 90 and a −/G polymorphism at position 119 (positions correspond to the ITS region of *B. dothidea* JN561088). It was confirmed that C_90_ and G_119_ were present in the strains of both genotype I and III, T_90_ and gap_119_ were present in the strains of genotype II, and C_90_ and gap_119_ were present in the genotype IV strain. This result indicated that the four genotypes of *B. dothidea* determined by introns can be also partially differentiated by ITS polymorphisms.

## Discussion

There have been no previous reports or descriptions of group I introns in *Botryosphaeria* species. In our study, however, four types of insertions were found at three different sites of the nuclear SSU rDNA in *Botryosphaeria dothidea*. In spite of their significant structural discrepancy, all the SSU insertions were identified as group I introns. Distribution of these introns among the 37 *B. dothidea* strains provided clear evidence of genetic diversity within this species.

Among the four introns, both Bdo.S943 and Bdo.S1506 were found to belong to subgroup IC1 with the typical characteristics of group I intron, namely, size ranging from 250–500 nt and approximately ten paired elements [36]. However, the 1349-nt Bdo.S1199-A differs from the general group I introns because it contains a large 848-nt ORF encoding a His-Cys HE, the main role of which is to mediate horizontal mobility of host introns at the DNA level, a process called “homing” [37]. This enzyme functions by recognizing and cleaving a specific 15- to 45-nt target sequence at or near the intron insertion site of an intron-less allele, and then the intron-containing allele is used as the template for inserting and reconnecting the broken double strand [38], [39]. The pattern promotes group I introns to spread within the homologous sites among populations of the same species. Nevertheless, according to the model proposed by Goddard and Burt [40], once the introns become fixed in one population, HEGs will gradually degenerate and finally be lost because of their redundancy of function. Therefore, we speculate that Bdo.S943 and Bdo.S1506 lost their HEGs after completely occupying the targeted populations, and that Bdo.S1199-B with the degenerate HEG is evidently an intermediate of the process whereby Bdo.S1199-A may eventually evolve into an intron lacking HEG.

Before using the above group I introns as markers to evaluate intraspecific genetic diversity, it is necessary to verify that each of the three introns (except Bdo.S1199-B, the descendant of Bdo.S1199-A) already existed in the ancestral *B. dothidea* so that they could co-evolve with all the other inherent genes of this species. This is necessary because a unique mechanism of group I introns, called “reverse splicing”, could enable group I introns to spread laterally among unrelated species, which may lead to erroneous interpretation of genetic structure of populations. Our sequence analysis results indicated that all three group I introns had homologs at the corresponding insertion sites in *N. dimidiatum*, a close relative of *B. dothidea* within the same family (Botryosphaeriaceae). Consequently, these introns share a common ancestor and they are relatively conserved in evolution, even though we do not yet know their rate of replication or rate of invasion of species or populations. On the other hand, group I introns are considered to be neutral or slightly deleterious to the host’s fitness thanks to the mechanism of self-splicing, which can help them release from pre-mRNAs and make the subsequent translation process proceed normally [37]. Therefore, group I introns are neutral in selection, and are suitable as markers of genetic diversity in populations.

Given the fact that an irregular distribution of the four group I introns was observed among the 37 isolates of *B. dothidea* surveyed, the most reasonable explanation is a cyclical loss and regain of introns among populations over the course of evolution [36]. A similar phenomenon has been reported in *Exophiala dermatitidis* and *Phialophora verrucosa* [6], [7]. In *E. dermatitidis*, the invasive genotype A and B strains from the natural environment were differentiated by the distribution of ribosomal introns. In *P. verrucosa*, 34 strains were classified into five genotypes according to intron distribution, and some correlation between genotypes and geographic location was inferred. For *B. dothidea*, four genotypes were established according to the patterns of intron distribution. Distinct from the other three types, genotype III was found only in Switzerland, which may have resulted from its evolution in a geographically isolated environment. Compared with genotype I and genotype II with a total prevalence of 95%, the fact that genotype III contains only one strain suggests a non-dominant population in China. The observation that genotype II strains were isolated only from apple and pear whereas genotype I strains had a wider host range (except pear) implies that genotype I may represent a dominant population. However, the hypothesized relationship between intron distribution and plant host or geographic origin requires testing with more data; in particular, size and geographic coverage of the sample need to be expanded.

Our findings are meaningful because they provide clues to patterns of evolution and distribution of group I introns in *B. dothidea*, a plant pathogen of substantial economic importance worldwide. We have also advanced efforts to comprehend the range of genetic diversity in this species. By using the introns as a marker, we can begin to understand the population structures of the species as well as the dominant population in a particular geographic area, which may give us some guidance to devise more efficient disease management. Second, these introns with variable distribution can be regarded as clues to analyse the evolutionary relationship among populations of *B. dothidea*. Because group I introns were speculated to be universal in related species of *B. dothidea*, they may be useful as tools to explore phylogenetics within this lineage. Moreover, some novel ideas might be proposed to control this kind of pathogens by targeting the introns. For example, drugs like pentamidine or 5-ﬂuorocystosine and antisense oligonucleotides that specifically inhibit the self-splicing of introns and kill intron-containing fungal strains have been developed [41], [42].
